# A Case of Cerebral Air Embolism After Dental Procedure

**DOI:** 10.7759/cureus.34976

**Published:** 2023-02-14

**Authors:** Filipa David, João S Castedo, Inês Prisco, Sara França, Celina Gonçalves, Sofia Monteiro

**Affiliations:** 1 Department of Internal Medicine, Hospital Pedro Hispano, Matosinhos Local Health Unit, Matosinhos, PRT; 2 Department of Anaesthesia and Hyperbaric Medicine, Hospital Pedro Hispano, Matosinhos Local Health Unit, Matosinhos, PRT; 3 Department of Neuroradiology, Hospital Pedro Hispano, Matosinhos Local Health Unit, Matosinhos, PRT; 4 Department of Neurology, Hospital Pedro Hispano, Matosinhos Local Health Unit, Matosinhos, PRT; 5 Department of Emergency and Intensive Care Medicine, Hospital Geral de Santo António, University Hospital Centre of Oporto, Oporto, PRT; 6 Department of Emergency and Intensive Care Medicine, Hospital Pedro Hispano, Matosinhos Local Health Unit, Matosinhos, PRT

**Keywords:** coma, dental procedure, super-refractory status epilepticus, hyperbaric oxygen therapy, cerebral air embolism

## Abstract

Air embolism is a rare and life-threatening event that occurs when air enters the cardiovascular system, usually secondary to iatrogenic vascular procedures. We present a 58-year-old woman who underwent a dental procedure (devitalization of a tooth) under local anesthesia, with a sudden onset of coma during manipulation and documentation of air in the vessels of the right frontal convexity sulci. After cerebral air embolism was confirmed, she received hyperbaric oxygen therapy, with resorption of the gas, but clinically she developed a super-refractory status epilepticus with a persistent coma. The slow clinical course required the exclusion of other etiologies of coma. The pathophysiology is not well known; however, it appears to be related to the injection of air by the high-speed dental drill through the soft tissue adjacent to the roots of the teeth, nearby the bloodstream. We highlight this event because of this unlikely association, which may delay diagnosis and the good results of hyperbaric medicine on prognosis.

## Introduction

An air embolism is a rare but potentially catastrophic event that occurs as a consequence of air entering the blood vessels, in the venous or arterial territory, with consequent vascular occlusion. The leading causes of air embolism are surgical manipulation, vascular procedures (including central line and cardiac catheterization), trauma or barotrauma, and regardless of the origin, at some point, a pressure gradient is required to drive air into the vascular system [1]. The incidence is unknown due to a large number of unreported and misdiagnosed cases, but recently the number of cases has increased along with the expansion of invasive procedures [1,2].

Air embolism mainly manifests with cardiovascular, pulmonary and neurological sequelae. In cerebral air embolism, the most commonly reported neurological symptoms are a sudden decreased level of consciousness with possible onset of coma, stroke-like focal deficits and seizures [3].

In this clinical case, we present a patient with cerebral air embolism after a devitalization of a tooth, highlighting the rarity of this complication in this context, the clinical severity and unexpected evolution, and emphasizing the emergency in early recognition and hyperbaric oxygen therapy (HBOT) as part of acute treatment with great impact on prognosis [1,4].

## Case presentation

We present a 58-year-old woman, with a medical history of controlled arterial hypertension and a smoker, who was submitted to an elective devitalization of tooth 21 under local anesthesia. During the dental manipulation, a sudden decreased level of consciousness was observed. The pre-hospital emergency team was activated and found the patient unconscious, scoring three on the Glasgow Coma Scale, therefore orotracheal intubation was performed to maintain a patent airway and she was transferred to a hospital with level III care.

**Figure 1:**
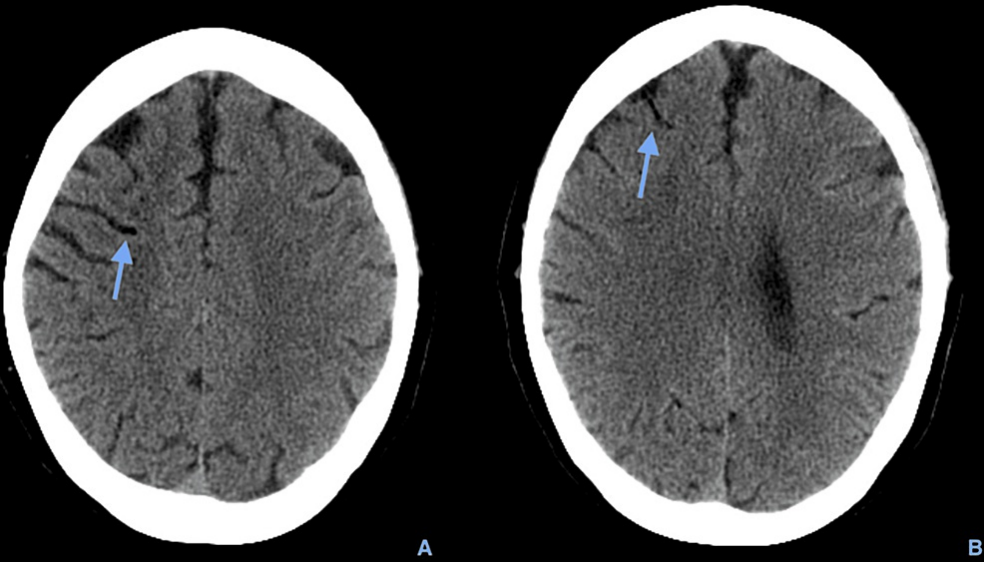
Axial brain CT scan showing serpiginous hypodensity in the high right anterior frontal high convexity (blue arrow on A and B), compatible with air in the sulci, with no ischemic lesion objectified

In the emergency room, the patient evolved with hemodynamic instability and point-of-care ultrasonography was performed by an intensive care team, showing a transient right ventricular dysfunction. On the presumption of a pulmonary embolism, thrombolysis with alteplase was performed, which was quickly discontinued after the exclusion of pulmonary embolism by thoracic computed tomography (CT) angiography. After hemodynamic stabilization, the patient realized a brain CT scan, which showed serpiginous hypodensity in the high right anterior frontal high convexity, compatible with air in the sulci, with no ischemic lesion objectified (Figures 1A, 1B).

**Figure 2:**
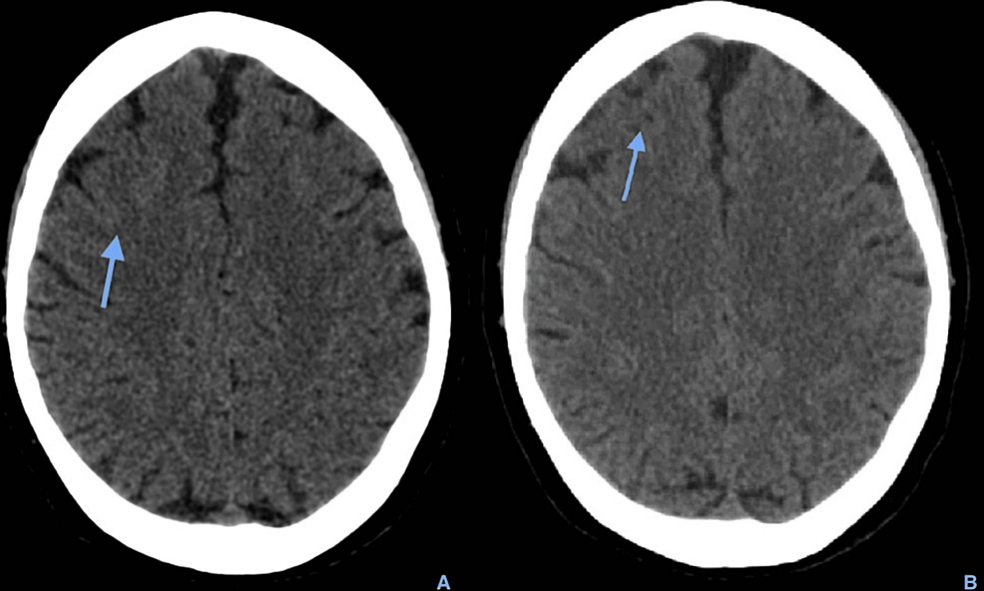
Axial brain CT scan five hours after HBOT therapy showing reabsorption of air at the anterior frontal high convexity sulci (blue arrow on A and B)

A diagnosis of cerebral air embolism was made and, given the extensive neurological involvement of the event, HBOT was performed five hours after the critical episode. During the procedure, the patient remained on invasive mechanical ventilation receiving sedation with propofol, fentanyl, and a neuromuscular blockade with rocuronium, under invasive monitoring and respiratory gas quantification. The protocol consisted of one session of HBOT with US Navy Table six protocol, maximum pressure of 2.8 absolute atmospheres and a total time of 4h45, which occurred without complications and with complete reabsorption of the gas embolism (Figures 2A, 2B).

Despite the imagological improvement, clinically the patient remained in a persistent comatose state. After intensive care unit admission, serial electroencephalograms were performed showing bi-hemispheric epileptiform activity with a predominant electrical discharge in the left frontal lobe, with no evidence of electroencephalographic or clinical improvement despite therapy, confirming the diagnosis of status epilepticus. The patient remained in this condition for 16 days while she was being treated with four periods of pharmacological burst-suppression and multiple optimized anti-seizure medications (levetiracetam, valproic acid, lacosamide, ketamine, thiopental) and it was considered a super-refractory status epilepticus.

**Figure 3:**
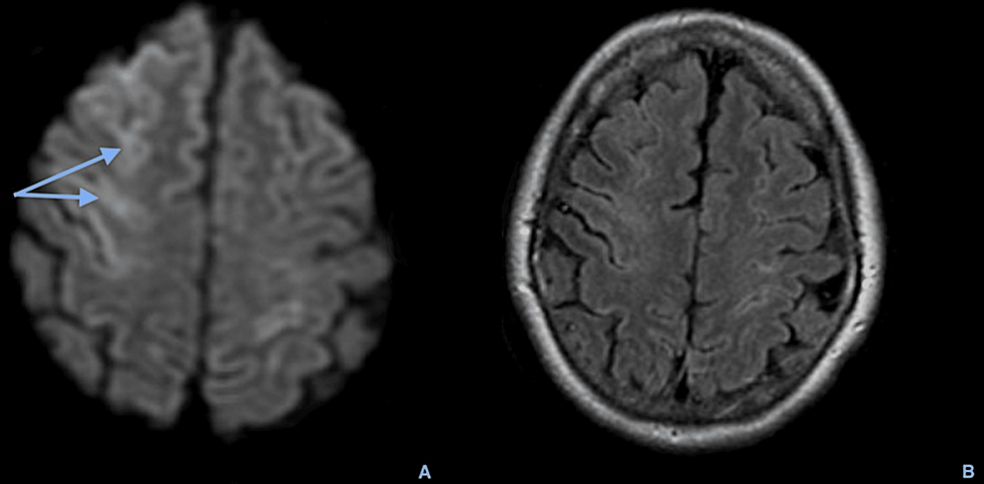
Axial brain magnetic resonance imaging, the first 24 hours after the event, showing on DWI a gyroscopic hypersignal in multiple cortical areas on the right frontal high convexity, consistent with a predominant cortical ischemic lesion (double blue arrow on A), still without translation on FLAIR (B) as expected in these lesions with a short time of onset

In addition, she underwent several brain magnetic resonance imaging (MRI) scans, in the first 24 hours after the event, which showed on DWI a gyriform hypersignal in multiple cortical areas on the right frontal high convexity, consistent with a recent predominant cortical ischemic lesion (Figures *3A, 3B*).

Two weeks after the event, the patient no longer had the criteria for status epilepticus; however, she had severe encephalopathy promoting a comatose state. The clinical course was very slow and no other disorders causing or perpetuating the coma were identified. After 60 days of hospitalization, there was a gradual improvement in the recovery of consciousness and language, with deficits indicative of the ischaemic lesion, namely left upper limb plegia. She was discharged at the end of 90 days to a rehabilitation unit.

## Discussion

A cerebral air embolism is a life-threatening event that must be identified early to reduce morbimortality. Iatrogenic events secondary to vascular procedures are the most prevalent etiology of this condition.

It is essential to be fully aware of the techniques and their possible complications so that when the clinical onset is acute and coexists with vascular manipulation, clinical suspicion can be prompt [4]. An emergency brain CT scan is essential for early diagnosis and management, with high sensitivity imaging technique, and air usually has a predilection for the supratentorial compartment with curvilinear hypodensities in the cerebral cortical vessels [2].

The exact pathophysiological mechanism of cerebral air embolism secondary to a dental intervention is not well known; however, since there is no description of any obvious relationship with local anesthesia, most authors agree that it is related to the use of dental instrumentation, namely the injection of air by the high-speed dental drill through the soft tissue adjacent to the roots of the tooth and therefore to the neurovascular tissue, which has contact with the bloodstream [4]. This increase in pressure, which is higher than the intravascular pressure, will allow air to enter the bloodstream and, in our case report, will justify the presence of air in the grooves of the right frontal convexity. There are very few reported clinical cases of cerebral air embolism caused by a dental procedure, and depending on the affected territory, venous or arterial, the literature proposes different pathophysiological mechanisms with distinct imaging findings. In a venous cerebral air embolism, we would have a retrograde increase of air in the systemic venous circulation, particularly in a vertical position. The typical imaging pattern of this venous event is serpiginous air density along the cortical sulci, particularly in the frontal cortical area, with gyroscopic patterns of acute near-air infarction, as was described in the case of our patient. In a cerebral arterial air embolism, and integrating with our case report, venous air from the pterygoid plexus draining into the superior vena cava and subsequently into the right cardiac cavities, could enter the cerebral arterial circulation. This may occur when the volume of air introduced exceeds the capacity of the pulmonary filter and, in the presence of a right-to-left intra-cardiac shunt or even an intra-pulmonary shunt, this air may reach the left cardiac cavities, passing through the aorta, right carotid artery, and finally access the right anterior and middle cerebral circulation. Arterial cerebral air embolism typically presents as punctate air densities in the cerebral parenchyma [4,5].

Despite the uncertainty of the air course, in the case described the images presented would be suggestive of a retrograde flow mechanism; on the other hand, although an intracardiac shunt has been excluded, the absence of a pulmonary shunt cannot be excluded, and the second mechanism may have taken place, explaining a pulmonary embolism phenomenon, which would be the explanation for the transient right heart instability and, subsequently, the cerebral air embolism manifesting with coma.

After the diagnosis, the priority is to prevent migration of the gas embolism to other sites and provide oxygen therapy with a maximum fraction of oxygen inspiration, while providing ongoing supportive care according to the clinical presentation [1,3,6]. The role of Hyperbaric Medicine in cerebral air embolism may be considered for more severe cases, such as major neurological changes and hemodynamic instability showing good results [7]. HBOT, based on two physics laws, decreases air emboli's size in two different ways. First, as described in Boyle's Law, the pressure exerted on the air bubble in a liquid environment will promote its reduction; second, the increase in partial pressure of the oxygen therapy supplied promotes the reabsorption of nitrogen gas from the bubble into the blood, creating high diffusion gradients, and also reducing the size of the air embolus, as described in Henry's Law [1,6,8]. Furthermore, this procedure induces cerebral vasoconstriction reducing cerebral edema as well as the probability of cerebral reperfusion injury and inflammation by inhibiting neutrophil endothelial adhesion [6]. Some retrospective studies show a greater benefit of HBOT in the first 6 hours after the event, producing better results, however, a period of up to 30 hours may be considered [1,6,8-9].

The persistence of coma, even when the patient no longer had epileptiform brain activity on electroencephalography, and with an MRI describing a single ischemic lesion with perilesional edema involvement of the right frontal lobe, led to further consideration of other factors perpetuating the coma, with no other causes identified. Slow recovery of wakefulness allowed the identification of focal motor deficits consistent with a territory of ischemic brain injury. This location is also very suggestive of the site where the gas bubbles were initially identified, and their extension is perfectly proportional to the severity of the event.

The status epilepticus was treated with pharmacological burst suppression protocols and anti-seizure medication and its electrical activity were more pronounced on the contralateral side of the embolus. Although a left frontal lobe lesion was not identified by conventional MRI, the presence of epileptiform activity at this site reflects a presumed focal impairment, and a bi-hemispheric involvement would allow a better understanding of the persistence of coma following the disappearance of epileptiform brain activity. It is also known that with an epileptiform activity that is sustained over time, the brain creates neuronal networks capable of propagating electrical activity to other cerebral areas, as may have occurred. After this event with severe neurological manifestations, the presence of a transient encephalopathy is expected.

Although the clinical impact of cerebral air embolism is variable, based on a prospective study that followed up to one year after diagnosis of iatrogenic air embolism for which all patients underwent HBOT, advanced age and delay in this treatment (>7h) were predictors of a worse clinical course with more neurological sequelae, as well as a lower Glasgow Coma Scale on admission and a long-term coma under mechanical ventilation. The last two conditions were present in our patient, which could justify a later recovery [10].

## Conclusions

As we know, with the number of invasive or minimally invasive techniques increasing over the years, the rate of new complications is also being reported. Cerebral air embolism has been one of those complications, associated with different techniques, and can be a catastrophic and even fatal event. This case aims to highlight cerebral air embolism as a rare but impactful complication of regular dental intervention. By knowing this uncommon association, healthcare professionals will be able to identify it earlier, and more importantly, to adopt a therapeutic approach with a positive impact on prognosis. HBOT as early treatment is highly beneficial in minimizing morbidity and mortality. We also stress the need to increase awareness of the severity of these events with a tremendous bi-hemispheric impact, which may result in persistent comatose states that are difficult to manage, even when detectable air bubbles are restricted to a focal area of the brain as described.
